# FcεRI Signaling in the Modulation of Allergic Response: Role of Mast Cell-Derived Exosomes

**DOI:** 10.3390/ijms21155464

**Published:** 2020-07-30

**Authors:** Mario Lecce, Rosa Molfetta, Nadia Domenica Milito, Angela Santoni, Rossella Paolini

**Affiliations:** 1 Department of Molecular Medicine, “Sapienza” University of Rome, Laboratory Affiliated to Istituto Pasteur Italia—Fondazione Cenci Bolognetti, Viale Regina Elena 291, 00161 Rome, Italy; mario.lecce@uniroma1.it (M.L.); rosa.molfetta@uniroma1.it (R.M.); nadia.milito@uniroma1.it (N.D.M.); angela.santoni@uniroma1.it (A.S.); 2 Istituto di Ricovero e Cura a Carattere Scientifico IRCCS Neuromed, 86077 Pozzilli, IS, Italy

**Keywords:** innate immunity, inflammation, immunoregulatory receptor crosstalk

## Abstract

Mast cells (MCs) are immune cells that act as environment resident sentinels playing a crucial role in Th2-mediated immune responses, including allergic reactions. Distinguishing features of MCs are the presence of numerous cytoplasmic granules that encapsulate a wide array of preformed bio-active molecules and the constitutive expression of the high affinity receptor of IgE (FcεRI). Upon FcεRI engagement by means of IgE and multivalent antigens, aggregated receptors trigger biochemical pathways that ultimately lead to the release of granule-stored and newly synthesized pro-inflammatory mediators. Additionally, MCs are also able to release exosomes either constitutively or upon stimulation. Exosomes are nanosized vesicles of endocytic origin endowed with important immunoregulatory properties, and represent an additional way of intercellular communication. Interestingly, exosomes generated upon FcεRI engagement contain co-stimulatory and adhesion molecules, lipid mediators, and MC-specific proteases, as well as receptor subunits together with IgE and antigens. These findings support the notion that FcεRI signaling plays an important role in influencing the composition and functions of exosomes derived by MCs depending on their activation status.

## 1. Introduction

Mast cells (MCs) are important components of the innate immune system and are implicated in a wide array of functions playing a crucial role in Th2 responses [1,2,3].

They represent a highly heterogeneous cell population whose phenotype and function are shaped by surrounding stimuli and growth factors [4,5]. The great diversification of MCs is especially evident from the vast range of receptors on their surface [6]. In this regard, all MCs constitutively express the high affinity receptor for IgE (FcεRI) whose signaling potently promotes MC effector functions in terms of degranulation and cytokine production [7,8]. This plethora of secreted molecules enable MCs to orchestrate physiological and pathological processes including adaptive immune responses, since they are able to cooperate with T and B lymphocytes and dendritic cells (DCs) [8,9,10,11]. Moreover, this important functional network is amplified by the release of biological active extracellular vesicles (EVs) that are able to deeply rewire the functional capability of recipient cells thanks to their delivered cargo [12,13,14]. Notably, EV composition is influenced by the kind of stimuli that MCs receive, thus depending on the microenvironment, MC-derived vesicles can have distinct functions.

This review will discuss evidence from the current literature about the multiple and apparently contrasting roles of MC-derived EVs in regulating Th2-associated immune responses with a focus on how FcεRI cross-linking affects the secretion rate and molecular composition of nanovesicles.

## 2. MC Subsets and Heterogenous Functional Specialization

MCs are granulated tissue-resident sentinel cells, which play a major role in responses against helminths and in type I hypersensitivity reactions including anaphylaxis [1,2]. However, they display a plethora of both physiological and pathological roles that also include the regulation of tumor behavior either promoting or preventing cancer progression [15,16,17].

Although MCs have been thought to derive from bone marrow hematopoietic stem cells, dual developmental MC origins have been recently proposed: most skin MCs are of primitive origin during embryogenesis (i.e., yolk sac derived) while adult MCs are of definitive origin (i.e., hematopoietic stem cell derived) [18,19]. Regardless, MC precursors undergo final maturation mainly under the influence of stem cell factor (SCF) and interleukin-3 (IL-3) [4,10,20,21].

MCs are disseminated through the whole body populating all vascularized tissues, and their presence is particularly abundant in organs and surfaces exposed to the external environment such as skin, gastrointestinal tract, and airways. Moreover, they are often placed in close connection with blood vessels, nerves, and smooth muscle cells. This widespread localization of MCs appears to result from constitutive homing, driven by adhesion molecules and chemokine receptors, and it is enhanced by inflammatory conditions [20,21,22,23]. Of note, the replenishment of adult tissue murine MCs predominantly arises from long-lived, tissue-resident precursors [18,19].

MCs have been canonically divided in two main subsets depending on tissue localization and granular protease content [4,5]. Murine mucosal MCs express chymases (mMCP-1 and mMCP-2) and usually populate the epithelial layer lining the surfaces of lung and intestinal tissues. The second subset is represented by connective tissue MC whose granules contain chymases (mMCP-4 and mMCP-5) and tryptases (mMCP-6 and mMCP-7) and have been found in skin and serosal cavities. Similarly, human MCs have been grouped into two categories: tryptase-only MCs (MC_T_), found mainly in mucosal tissues such as airways and lungs that phenotypically resembles murine mucosal MC; and MCs positive for tryptase and chymase (MC_TC_), that in analogy to murine connective tissue MCs are located particularly in skin, gastrointestinal tract, and conjunctiva [4,5,24]. However, this classification is now considered not completely exhaustive since a large amount of evidence has shown that MCs represent a highly plastic cell population whose phenotype and effector functions may be profoundly shaped by the surrounding microenvironment [25,26,27,28]. For example, it has been observed that the presence of CD25 discriminates two different groups of MCs relying on transcriptional profile and capacity to respond to certain stimuli [28]. Moreover, MCs with a connective tissue phenotype when located in different organs such as lung and intestine exhibit slightly different tryptases and chymases composition [27]. 

Related to this, a transcriptional profiling of murine MCs unveiled a remarkable variability with significant differences of gene expression signature depending on the tissue examined [29]. Notably, a global proteome profiling of both human and mouse connective tissue MCs revealed a unique protein pattern far away from other immune cells even from that of the closely related granulocytic lineage [30].

One of the most distinguishing morphological features of MCs is represented by MC granules that occupy almost the entire cytoplasm, appear electron-dense, and contain considerable amounts of bioactive molecules ready to be released upon MC stimulation. They encapsulate various mediators such as proteases, cytokines, chemokines, amines (e.g., histamine and serotonin), and enzymes including the β-hexosaminidase [31,32].

Both in physiology and pathology, MC immunoregulatory functions critically depend on these compounds that can shape the phenotype and activity of surrounding immune cells orchestrating their responses [8,33].

## 3. FcεRI-Mediated Mast Cell Signaling

MC degranulation and effector functions are finely controlled by an elevated number of cell surface receptors that enable MCs to sense and respond to a plethora of microenvironmental stimuli [3,6]. One of the most studied is the high affinity receptor for IgE, namely FcεRI. Structurally, it is composed of one α-chain bearing two extracellular immunoglobulin domains that bind to the Fc portion of a single molecule of IgE, a β-chain that holds an immunoreceptor tyrosine-based activation motif (ITAM), and a homodimer of disulfide-bound ITAM-containing γ-chains [7].

The recognition of multivalent antigen by FcεRI-bound IgE triggers a complex biochemical pathway initiated by the phosphorylation of ITAM motifs by the Src family kinase Lyn. Phosphorylated ITAMs function as docking sites for the Syk kinase that plays a crucial role in the propagation of downstream signaling by phosphorylating many signaling molecules and in concert leading to degranulation and subsequent immediate release of diverse bioactive molecules [34].

FcεRI proximal signaling parallelly includes a Fyn kinase-driven pathway that plays an important role in potentiating phospho-inositide 3-kinase (PI3K) activation [35]. Of note, Fyn also controls Ca^2+^ influx allowing the transit of calcium when the endoplasmic stores are depleted [36].

Besides multivalent antigens, the receptor also responds to monovalent antigens that can initiate signaling through the passive exclusion of the tyrosine phosphatase CD45 from the vicinity of membrane regions of the aggregated FcεRI complexes [37]. Thus, this form of receptor triggering may extend the reactivity of MCs to antigens in vivo.

FcεRI engagement can also induce negative signals through the combined action of protein and lipid phosphatases as well as ubiquitin ligases that limit the extent and duration of activating signals [38,39]. Of note, ubiquitination of engaged receptors and associated kinases generates signals for the delivery of internalized ubiquitinated receptor complexes to lysosomes for degradation, providing negative-feedback regulation of receptor activity [38].

When positive signals prevail, granule exocytosis is finally triggered by inositol-1,4,5-trisphosphate (IP3) that increases cytoplasmatic Ca^2+^ levels and induces the subsequent activation of protein kinase C (PKC), finally leading to the recruitment of the degranulation machinery. In particular, V-SNARE and t-SNARE proteins play a critical role in controlling degranulation activity: they are positioned on the surface of granule and on the plasma membrane, respectively, and their interaction is required for granule fusion and the consequent release of stored mediators [34].

Within a few hours of FcεRI stimulation, several transcription factors including NFAT, NFκB, and AP-1 are activated and stimulate the transcription of a high number of genes including those encoding pro-inflammatory cytokines (e.g., TNF-α, IL-6, and IL-13) and responsible for the late phase of allergic responses [2,34,40].

Of note, in the past few years, a growing number of reports showed that MCs also communicate with neighboring and distant cells through the release of extracellular vesicles (EVs) both constitutively and during FcεRI-induced degranulation [12,13,14,41].

## 4. Extracellular Vesicles: Biogenesis and Composition

**Figure 1:**
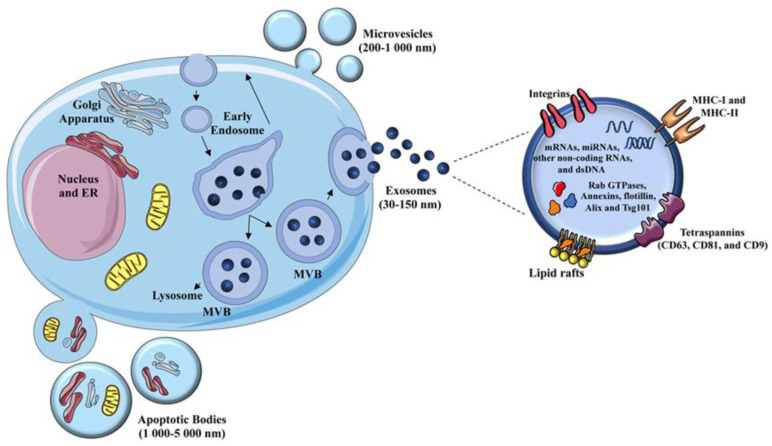
Biogenesis and composition of extracellular vesicles. Extracellular vesicles (EVs) include microvesicles, exosomes, and apoptotic bodies. Microvesicles are shed from the plasma membrane through protrusion or budding; exosomes are nanovesicles released from specific endosomal compartments, namely Multivesicular Bodies (MVB), upon their fusion with the plasma membrane; apoptotic bodies are generated by blebbing of the plasma membrane from cells undergoing apoptosis. Exosomes have been studied most widely, and their components, including lipids, proteins, and nucleic acids, are depicted. Most of them are shared with microvesicles. Modified from [42].

EVs include a heterogenous group of membrane-bound particles present in all biological fluids that could be broadly grouped, based on their size and origin, in micro- and nanovesicles also called exosomes (Figure 1) [42].

EVs represent an important vehicle of intercellular communication and immune regulation, able to rewire and modulate effector functions of target cells. Indeed, EVs can transfer their packaged cargo that include lipids, proteins, and nucleic acids derived from the donor cells, thus modifying target cell composition and function [43,44,45].

Regarding the size, microvesicles are characterized by a diameter ranging from 200 to 1000 nm and originate from an outward budding and subsequent pinch off of the plasma membrane. Nanovesicles or exosomes have a smaller diameter (30–150 nm) and are characterized by an endosomal origin.

Both microvesicles and exosomes are generally described as EVs derived from healthy cells. However, damaged dying cells, including transformed cells, can also release a variety of large membrane-bound vesicles (1000–5000 nm) broadly known as apoptotic cell-derived EVs (Figure 1) [46]. Most studies have focused on microvesicles and exosomes released from healthy and cancerous cells, while the roles of apoptotic cell-derived EVs remain underexplored.

Various molecular components are required for EV biogenesis and release [47,48,49,50,51].

As an initial step, an important role for microvesicle release is played by clusters of lipids and proteins on the plasma membrane that gather together in order to generate microdomains. Then, a machinery involved in the rearrangements of lipids composed of scramblase, flippase, and floppase is responsible for contributing to the asymmetry of phospholipids that promotes membrane curvature. The final step consists of the budding and formation of microvesicles thanks to the contribution of proteins involved in cytoskeleton rearrangement such as ARF6 [49].

Exosome formation comprises multiple steps, closely related to the biogenesis of multivesicular bodies (MVBs), endosomal compartments characterized by the presence of multiple intraluminal vesicles (ILVs). One of the most studied mechanisms involves the endosomal sorting complex required for transport (ESCRT) machinery that enables endosome membrane curvature and invagination with the subsequent ILV formation within MVBs [52]. ESCRT complexes are composed of ESCRT-0 and ESCRT-I that promote cargo recruitment, while ESCRT-II and ESCRT-III, together with numerous accessory proteins, induce the inward budding of endosomal membrane and pinch off of ILVs. Notably, Hrs, a protein member of the ESCRT-0 complex, is critically involved in exosome biogenesis since its depletion on DCs, MCs, and HeLa cells strongly impairs nanovesicle secretion [51,52,53]. The ESCRT machinery also serves to ensure the proper composition of the vesicles. Indeed, a study of carcinoma cell line Hep-2 found that the absence of ESCRTs resulted in impaired cargo sorting into ILVs [54].

Several studies have shown that multiple ESCRT-independent lipid-driven mechanisms can be involved in MVB formation and exosome biogenesis [47,48,54]. In particular, the rat basophilic cell line RBL-2H3 expressing an inactive form of phospholipase D2 (PLD2) showed reduced exosome production with respect to cells expressing the active enzyme, supporting the involvement of PLD2 in exosomes biogenesis [47]. Moreover, sphingomyelinase 2, an enzyme responsible for the conversion of sphingomyelin in ceramide, is implicated in exosome formation, since its inhibition strongly decreases nanovesicle production [48].

The major components of exosomes, depicted in Figure 1, include numerous proteins normally found in endosomes such as Rab GTPases, Annexins, flotillin, Alix, and Tsg101, reflecting the composition of the compartment where they are generated [55]. Tetraspannins such as CD63, CD81, and CD9 are commonly present in nanovesicles to such an extent that they are generally considered exosomal markers. Nanovesicles also contain lipids derived from plasma membranes (e.g., cholesterol, sphingomyelin, and phosphatidylserine), and to a minor extent, lipids encapsulated from the cytoplasm [56]. Interestingly, a heterogenous group of nucleic acids that includes mRNA, miRNA, tRNA, and dsDNA, appears to be selectively conveyed into nanovesicles [57,58,59], and is able to regulate phenotype and effector functions of several target recipient cells [55,58,59]. Indeed, once released into the extracellular milieu, exosomes can be specifically up-taken by neighboring cells or enter the circulation and exert multiple effects on cells located in distant sites [60,61]. They can stimulate intracellular signaling through receptor–ligand interaction or deliver their protein and RNA content upon internalization or fusion with the plasma membrane, thus modulating the biological functions of target cells [43,44,49,62].

In regard to their role during immune responses, body fluid exosomes are able to modulate cytokine response in monocytes through the regulation of gene expression [63], while exosomes derived from DCs, B lymphocytes, and tumor cells have been shown to mediate antigen presentation likely through the presence of preformed peptide-MHC class I/II complexes [55,64,65].

Regarding the protein cargos of microvesicles, they include vesicle-associated membrane protein 3 (VAMP3) that derives from the endosomal recycling pathway, but also MHC class I and II together with the β1 integrin receptor [55,66], supporting a role for microvesicles in the regulation of adaptive immunity. Furthermore, microvesicles can modulate immune responses by transporting cytokines such as IL-1β [67] and nucleic acids such as proinflammatory microRNAs [68].

Given the important roles of EVs and particularly of exosomes in various biological processes, great efforts have been made in recent years to introduce different methods able to isolate exosomes from diverse biological sources, including commercial kit- and microfluidics-based techniques [69]. Nowadays, the sequential ultracentrifugation-based method is considered the gold standard of exosome isolation. However, due to the heterogenous diameter of exosomes and the high possibility of size overlap with other EVs, it is always necessary to couple isolation methodologies with approaches able to estimate and compare sample purity and size distribution.

It is also important to consider and incorporate the recommendations from the Minimal Information for Studies of Extracellular Vesicles 2018 (MISEV2018) [70] when conducting exosome/EV-related experiments to enhance rigor and reproducibility.

### 4.1. Heterogeneity of MC-Derived EVs

MCs are endowed with massive granular compartments, representing one of the most important secretory cells of the immune system. Thus, it is not surprising that over the years they have emerged as a relevant producer of EVs empowered with clear regulatory functions including the modulation of immunological responses mainly to a direct targeting of DCs, lymphocytes, and other MCs, as detailed below [12,13,14].

Currently, most of the literature leads to a remarkable better dissection of MC-derived exosome functions in several contexts; conversely, the roles of microvesicles produced by MCs still remain little known. This may be attributable to the fact that specific attention has been devoted to the common compartment and pathway(s) involved in the biogenesis of exosomes and secretory granules since MC effector functions principally rely on granule exocytosis. Moreover, the release of pre-stored mediators through MC degranulation is often accompanied by exosome release, suggesting that there might be different types of vesicles inside MVBs [55,71].

Raposo et al., for the first time, described the presence of three different kinds of endosomal compartments within the cytoplasm of mouse BMMCs (mBMMCs) [71]: type I that contains EVs with morphological features resembling the exosome; type II that includes secretory granules containing serotonin; type III which is endowed with an electron dense core.

Through both constitutive and induced exocytosis by means of IgE and multivalent antigens, mBMMCs and rodent MC lines release exosomes that shuttle several mRNA and microRNA and also deliver MHC II molecules and FcεRI subunits [13,51,57,71,72,73].

**Figure 2:**
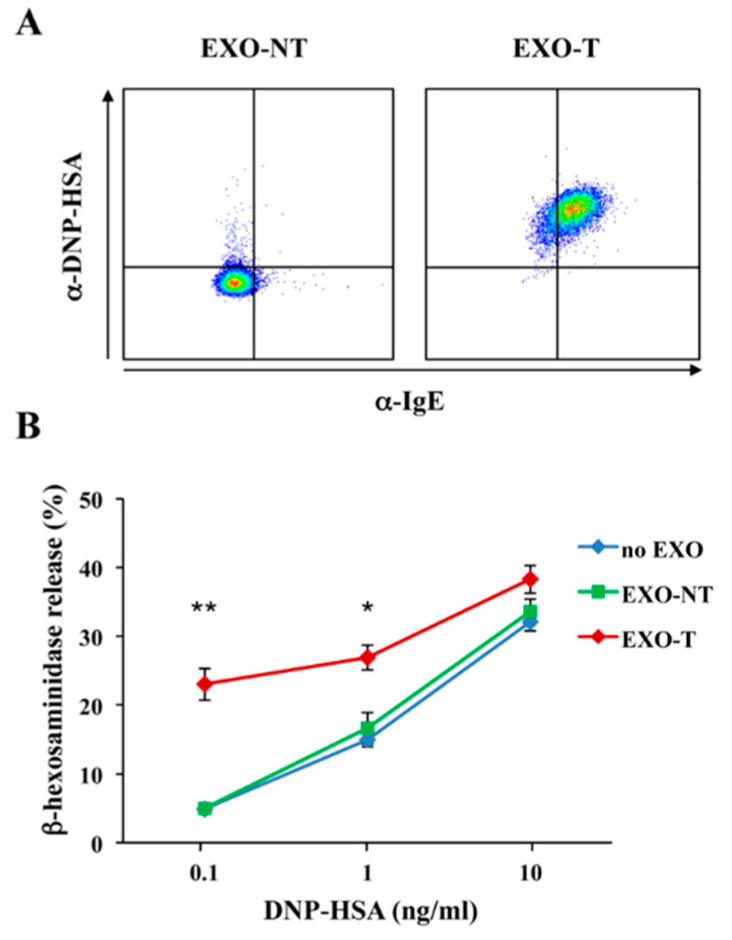
Mast cell (MC)-derived exosomes endowed with IgE and antigens cooperate with a soluble antigen to induce MC degranulation. (**A**) RBL-2H3 cells were sensitized with 1 μg/mL IgE (clone SPE-7) ON at 37 °C and then stimulated or not with 1 μg/mL DNP-HSA for 6 h. Exosomes (EXO) were purified by sequential ultracentrifugation from culture supernatants of unstimulated (EXO-NT) and stimulated (EXO-T) RBL cells, and then 4 μg of EXO-NT and EXO-T were passively adsorbed on 4 μm Aldehyde/Sulfate Latex Beads (Life Technologies, Carlsbad, CA, USA) ON at 4 °C. Exosome/bead complexes were washed and labelled with AlexaFluor 488 anti-DNP (Life Technologies, Carlsbad, CA, USA) and BV-510 anti-IgE (BD biosciences). (**B**) RBL-2H3 cells were sensitized by the addition of IgE for 1 h at 37 °C. IgE-loaded cells were then incubated with EXO-NT or EXO-T (20 μg/mL) together with the indicated suboptimal doses of DNP-HSA. After 30 min, cell culture supernatants were collected and β-hexosaminidase release was measured as marker of degranulation. As the control, β-hexosaminidase was analysed in exosome lysates that resulted negative (data not shown). All data are presented as the mean ± SD of three independent experiments. * *p* < 0.05, ** *p* < 0.01, one-way ANOVA with Tukey’s multiple comparison test. The technique used to isolate exosomes has been fully described in [51].

However, EVs generated upon FcεRI engagement are different in term of size, morphology, and molecular profiles from those released from unstimulated MCs: they contain co-stimulatory and adhesion molecules, lipid mediators as well as phospholipases (PLA2, PLC, PLD), and biologically active MC-specific proteases (carboxypeptidase A3, tryptase, chymase) [47,74,75,76], strongly supporting the hypothesis that activation status affects the type and the composition of released vesicles. Of note, upon antigen stimulation, we have recently reported an enrichment of receptor subunits on exosomes released by RBL-2H3 cells that also contain IgE and multivalent antigens [51] (Figure 2, panel A), suggesting that FcεRI-mediated signaling drives a selective sorting of the engaged receptor complexes into exosomes.

In this regard, we have previously reported in RBL-2H3 cells that upon antigen stimulation, FcεRIβ and γ subunits undergo ubiquitination, thus triggering receptor endocytosis from the cell surface to lysosomes for degradation [77,78] and envisaged a key role for the endocytic adapter Hrs [79]. More recently, we found that Hrs is required for exosome release by MCs, as well [51].

Thus, it is likely that exosomes originate from the same endocytic compartment in which receptor complexes are delivered. Whether ubiquitination is a signal required for receptor sorting into exosomes is still under investigation.

Given all these data, it is conceivable that MCs release EVs endowed with different functions and immunomodulatory activities based on the activation status, thus regulating inflammatory responses either in a beneficial or detrimental manner.

### 4.2. MC-Derived EVs in the Modulation of Inflammatory Responses

In regard to the potential contribution of MC-derived EVs in regulating the inflammatory response, several and apparently conflicting data exist, obtained from experimental animal models and using murine and human mast cell lines.

A recent report provides evidence that exosomes released from unstimulated mBMMCs contain FcεRI complexes able to bind to free IgE, thus decreasing circulating IgE levels and inhibiting allergic cascade [73]. However, there are several other lines of evidence that support a positive immune regulatory role for MC-derived exosomes [51,80,81,82]. The interaction of exosomes produced by unstimulated mBMMCs with naïve T cells results in their polarization towards the Th2 phenotype in vitro [82]. Moreover, BMMCs constitutively release hsp60- and hsp70-positive nanovesicles that enable DC phenotypic and functional maturation with subsequent increased expression of MHC class II molecules, CD80, CD86, and CD40 [81]. Additionally, P815 mastocitoma and the mast cell line MC/9 secrete nanovesicles capable of stimulating proliferation of murine spleen-derived B and T lymphocytes and T cell secretion of IL-2 and IFN-γ in vitro [80]. The mitogenic activity was also attributed to mBMMC-derived exosomes both in vivo and in vitro but only after MC pretreatment with IL-4 [80]. Of note, human MC-derived exosomes transfer PLA2G4D to neighboring cells thus contributing to a CD1-reactive T cell response in psoriasis individuals [76].

Little is known about the mechanisms underlying the ability of MC-derived exoxomes to activate other MCs and/or immune cells other than MCs. They could trigger intracellular signal cascade(s) by interacting with membrane-bound receptors and/or modulate target cell functions upon up-take and delivery of their specific cargo [60,61]. In this context, mBMMCs constitutively release nanovesicles that express hsp70 and are internalized by DCs thanks to the interaction with CD91 [81].

Interestingly, nanovesicles derived from activated mBMMCs or MC lines can be transferred to resting and/or other activated MCs potentiating their activation [51,57,74,75]. In particular, we found that the presence of IgE/antigen complexes on the exosome surface facilitates their up-take by IgE-sensitized RBL cells and mBMMCs through receptor dependent endocytosis, and triggers MC degranulation and cytokine production [51]. To extend these findings, we have investigated whether an antigen exposed on the exosome surface could cooperate with a soluble antigen to induce MC degranulation.

The addition of exosomes derived from stimulated RBL-2H3 cells (EXO-T) significantly increased β-hexosaminidase release induced by suboptimal doses of multivalent soluble antigen, while, exosomes produced by unstimulated MCs (EXO-NT) did not affect antigen-induced MC degranulation (Figure 2, panel B). Similar results were also obtained with primary mBMMCs (data not shown).

Whether this cooperative mechanism might operate in local responses to allergens and/or in systemic reactions is still unknown and needs further investigation.

Interestingly, human MCs also have the potential to deliver IgE/antigen complexes on exosomes since nanovesicles present in the sera of atopic donors expose FcεRI subunits and IgE on their surface [51].

**Figure 3:**
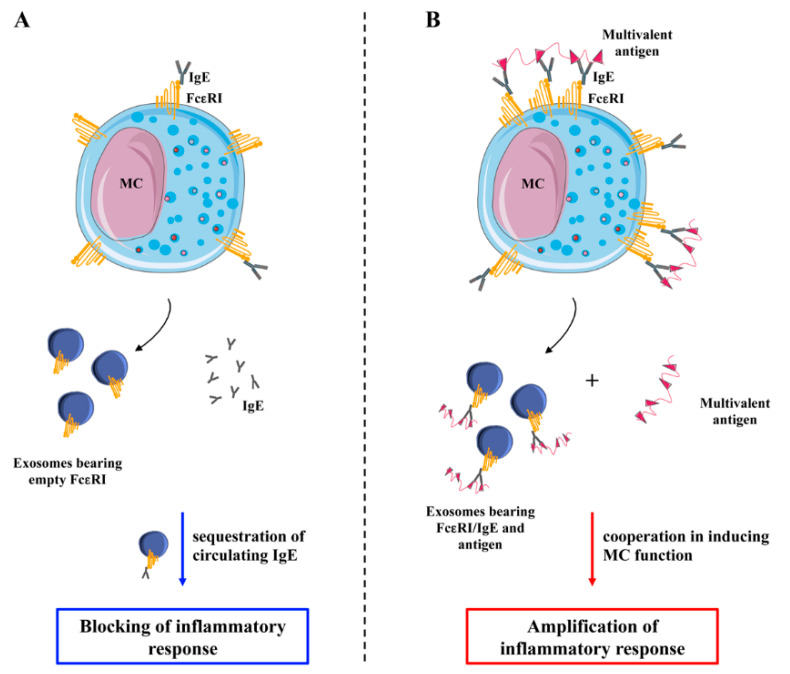
Modulation of IgE dependent inflammatory response by MC-derived exosomes. (**A**) MCs release exosomes bearing empty receptors either constitutively or upon antigen-independent stimuli that can bind free IgE inhibiting the inflammatory response. (**B**) Upon FcεRI engagement by means of IgE loading followed by antigen stimulation, MCs release exosomes bearing receptors, IgE, and antigens that can cooperate with soluble multivalent antigens to amplify the inflammatory response.

In this scenario, a likely model is the one depicted in Figure 3. MCs release exosomes bearing empty FcεRI complexes either constitutively or upon antigen-independent stimuli. These nanovesicles can bind free IgE and inhibit the inflammatory response (Panel A). On the other hand, upon FcεRI engagement by means of IgE and antigens, MCs release exosomes bearing receptors, IgE, and antigens that can cooperate with soluble antigens to stimulate other MCs, thus amplifying the inflammatory response (Panel B).

This mechanism may also represent a mean of antigen delivery and stimulation of FcεRI-positive immune cells other than MCs. In particular, exosome-mediated uptake of antigen by FcεRI positive DCs may potentiate their capability of antigen presentation and trigger antigen-specific T cell responses, further sustaining an allergic response.

In this scenario, pharmacological treatment able to inhibit MC degranulation could represent an interesting approach to affect MC exosome release and inflammation outcome. Of note, treatment with ketotifen, a MC stabilizer, dramatically reduces exosome secretion by cancer cells [83].

## 5. Concluding Remarks

MCs have recently captured considerable attention as an important source of EVs implicated in many physiological and pathological processes.

MC-derived EVs act as a vehicle for a wide variety of bioactive cargos preventing their degradation and allowing them to modulate the function of surrounding cells. Moreover, exosomes released by MCs upon antigen-mediated FcεRI engagement may be profoundly different from those released by unstimulated MCs in terms of composition and immune modulatory functions. Whether activating receptors other than FcεRI (e.g., toll-like receptors and MAS-related G protein-coupled receptor X2) may modulate EV secretion, composition, and functions represents a novel potential field of investigation.

In the context of an IgE dependent response, exosomes released by FcεRI-engaged MCs that contain FcεRI/IgE/antigen complexes may contribute to the spreading of information among MCs by allowing antigen persistency, and thus amplifying allergic reactions.

However, the contribution of different MC subsets to the production of EVs endowed with diverse immune modulatory functions is still rather unexplored.

A better understanding of how exosomes influence the hypersensitivity reactions could help to prevent allergies and lead to the development of more efficient engineered exosome-based therapies.
